# A Nominally Safe Dose of Fumonisins Induces Mild Neuroinflammation in Chickens by Targeting Sphingolipids and Oxylipins but Not Cytokines

**DOI:** 10.3390/antiox15050546

**Published:** 2026-04-25

**Authors:** Philippe Guerre, Elodie Lassallette, Didier Tardieu, Marie Berthommier, Alix Pierron Baysse

**Affiliations:** 1 IHAP, ENVT, INRAE, Université de Toulouse, 31076 Toulouse, France; elodie.lassallette@envt.fr (E.L.); didier.tardieu@envt.fr (D.T.); marie.berthommier_20@envt.fr (M.B.); alix.pierron@envt.fr (A.P.B.); 2 Olmix S.A., ZA du Haut du Bois, 56580 Bréhan, France

**Keywords:** fumonisins, brain, inflammation, sphingolipids, oxylipins, cytokines

## Abstract

Alterations in sphingolipids (SLs), oxylipins (OLs) and cytokines (CKs) are central to neuroinflammation. However, the effects of low doses Fumonisins B (FBs) on these analytes in the avian brain remain unclear.This study investigated SLs, OLs, CKs, and the activities of phospholipase A2c (PLA2c) and cyclooxygenase 2 (COX2) in the brains of chickens exposed to FB at a nominally safe dose of 14.6 mg FB1 + FB2/kg for 14 and 21 days. Targeted LC-MS/MS analyses revealed that FB exposure increased brain concentrations of sphingosine, N-acetyl-sphingosine, sphingosine 1-phosphate (So1P), ceramides (Cers), and sphingomyelins (SM). The Cer:SM ratio was elevated at 14 days but normalized by 21 days, whereas the So1P:Cer ratio rose at 14 days and continued to increase at 21 days. These changes coincided with elevated PLA2c and COX2 activities. OL profiling indicated a modest rise in pro-inflammatory arachidonic acid-derived COX metabolites at 14 days, while anti-inflammatory OLs derived from COX and lipoxygenase (LOX) pathways, including PGE2, 15-HETE, and 17-HDHA, increased significantly at 21 days. In contrast, the levels of CKs changed only slightly. Brain concentrations of Fumonisin B1 (FB1) indicated increased blood–brain barrier permeability.These findings highlight a key role of Cers in modulating OL production in FB neurotoxicity.

## 1. Introduction

Fumonisins B (FB) are widespread food contaminants whose toxicity has been thoroughly reviewed [1,2]. Approximately 70% of the world’s food crops, particularly corn and corn products, are reportedly contaminated with FB [3]. Although the discovery of FB is historically associated with the onset of equine leucoencephalomalacia (ELEM), most studies on FB toxicity have focused on its hepatotoxic, nephrotoxic, and carcinogenic effects [1,2,4]. With the exception of ELEM, most research on FB neurotoxicity has been conducted at relatively high doses or through direct administration into the brain or in vitro. These studies demonstrated that FB1 induces neural tube defects and that neurons and glial cells exhibit differential sensitivity to FB-induced toxicity [1,2,5,6]. However, comparative in vivo and in vitro experiments revealed that FB1 only crosses the blood–brain barrier (BBB) at high doses [7], and the neuroinflammatory effects of FB at realistic exposure levels remain largely undocumented.

Due to their structural resemblance to sphingoid bases (SB), FB toxicity primarily stems from the inhibition of ceramide synthase (CerS) [4]. This inhibition decreases ceramide (Cer) levels and elevates the sphinganine:sphingosine (Sa:So) ratio, as measured in SB and their phosphorylated forms. Additional biomarkers, including the C22–C24:C16 ratios are also affected [8]. SLs are key compounds in the brain, and several alterations in Cer, sphingomyelins (SMs), monohexosylceramides (HexCers), and lactosylceramides (LacCers) are involved in the onset and progression of neuroinflammatory and neurodegenerative diseases [9]. Interestingly, a study of chickens fed a nominally safe dose of FB for four or nine days revealed SL alterations that could not be solely explained by CerS inhibition [10].

Oxylipins (OLs), formed from the oxidation of polyunsaturated fatty acids (PUFAs) following phospholipase A2 (PLA2) hydrolysis of membrane glycerophospholipids, contribute significantly to neuroinflammatory processes [11,12]. Arachidonic acid (AA) and docosahexaenoic acid (DHA) are the primary PUFAs in the brain and represent the ω6 and ω3 classes, respectively [13]. PUFA oxidation by cyclooxygenases (COXs) generates prostaglandins, while lipoxygenases (LOXs) produce hydroxylated OLs. P450 enzymes generate epoxides and monohydroxylated OLs. Moreover, non-enzymatic (NE) oxidation occurs during oxidative stress and inflammation [13]. Generally, AA-derived OLs via COX can be pro- or anti-inflammatory, whereas LOX- or P450-derived OLs tend to be anti-inflammatory; DHA-derived LOX products are predominantly anti-inflammatory [13,14]. Interestingly, a recent study has revealed that several OLs derived from AA and DHA were increased in the brains of chickens fed FB [15]. However, the relationship between the effects of FB on OL and SL remains unknown.

In addition to SLs and OLs, cytokines (CKs) are crucial mediators of inflammation arising from brain injury [16]. TNFα, IL1β, and IL6 orchestrate early inflammatory responses, whereas IL-10 promotes resolution [17]. CKs also regulate metabolism: TNF induces insulin resistance, IL-1β stimulates insulin secretion, and IL-6 redistributes energy from storage to active tissues [18]. Pro-inflammatory cytokines activate PLA2c and sphingomyelinase, creating a feed-forward loop that exacerbates inflammation. CK involvement in FB neurotoxicity remains poorly characterized. One mouse study reported elevated TNFα, IL1β, IL6, and IFNγ in brain regions after intracerebroventricular FB1 administration [19], while in vitro glial studies showed decreased TNFα and IL1β [20]. Finally, although SLs, OLs, and CKs are all key regulators of inflammation, their roles and interactions in FB neurotoxicity have never been studied together.

The aim of this study was to clarify the inflammatory effects of FB by measuring SLs, OLs, CKs, and PLA2c/COX2 activity in the brains of chickens fed a nominally safe dose of FB. Targeted LC-MS/MS analysis was used to measure the concentrations of SLs and OLs in order to identify any quantitatively scarce analytes with strong biological properties. Various ratios and correlations were calculated to identify the metabolic pathways involved in the observed alterations and their interconnections. Measurements of FB1 in the brain were also taken to evaluate the diffusion of the toxin into the brain. These analyses were performed at two exposure times to determine how they evolved over time.

## 2. Materials and Methods

### 2.1. Reagents and Chemicals

All chemicals and reagents were obtained from Sigma-Aldrich Chimie SARL (Saint Quentin Fallavier, France) or Scharlab S.L. (Sentmenat, Spain). Reagents were of HPLC grade, except those used for LC-MS/MS analyses, which were LC-MS grade. FB standards and internal standards (IS) solutions, [13C34]-FB1, [13C34]-FB2, and [13C34]-FB3 were purchased from Biopure™ (Romer Labs, Getzersdorf, Austria). FUMONIPREP^®^ columns were supplied by R-Biopharm (R-Biopharm Rhone LTD, Glasgow, UK). Oasis HLB 3cc extraction columns were obtained from Waters™ (Milford, MA, USA).

SL external standards (Table S1) and ISs were obtained from Avanti Polar Lipids (Alabaster, AL, USA) and Sigma (Saint Quentin Fallavier, France). The IS corresponded to the “Ceramide/Sphingoid Internal Standard Mixture I” supplemented with m17:1/12:0, m18:1/12:0, and C12:0-ceramide sulfatide to obtain a final IS concentration of 6250 pmol/mL for each analyte. OL external standards (Table S2) and IS were obtained from Interchim (Montluçon, France) and corresponded to Cayman Chemical products (Ann Arbor, MI, USA).

The IS mixtures included primary COX and LOX MaxSpec^®^ LC-MS Mixture, Deuterated Arachidonic Acid CYP450 Metabolite MaxSpec^®^ LC-MS Mixture, and Deuterated Linoleic Acid Oxylipins MaxSpec^®^ LC-MS Mixture. The IS mixtures were combined (*v*/*v*/*v*) before extraction to achieve a final IS concentration of 1 µg/mL per analyte.

### 2.2. Feed and Animals

Animal experiments were conducted in compliance with the European Directive EC2010/63 for the care and use of animals in research (approval number V11941, project 2017062111426641, accepted by the French Ministry of Higher Education, Research and Innovation on 6 November 2017) [21]. Briefly, experimental diets were prepared by Tecaliman (Nantes, France) based on corn and soybean. The FB-contaminated diet was formulated by incorporating naturally contaminated corn to achieve final concentrations of 9.4 mg FB1/kg and 5.21 mg FB2/kg of feed. Mycotoxin-free corn was used for the control diet. A total of 41 mycotoxins were quantified by Labocéa (Ploufragan, France) using LC-MS/MS [21]. All but FB were not detected or were present only at trace levels. Thirty-one-day-old chickens were randomly allocated to three indoor pens with ad libitum access to feed and water at Cébiphar (Fondettes, France). After two weeks of acclimation, chickens were assigned to the following groups: control diet for 21 days (Con, n = 10), FB diet for 21 days (FB21, n = 10), and control diet for 7 days followed by FB diet for 14 days (FB14, n = 10). Feed intake and body weight were recorded on days 15, 21, 28, and 35. Chickens were sacrificed by electrical stunning (electronarcosis) followed by exsanguination over two consecutive days, with an 8 h fasting period prior to sacrifice. All animals underwent macroscopic examination for gross pathology, and brains were collected and stored at −80 °C until further analysis.

### 2.3. Extraction of Fumonisins, Sphingolipids, and Oxylipins

Fumonisins were quantified in five brain homogenates prepared from two animals per group, as previously described [10]. Briefly, 1 g of brain tissue was homogenized using an Ultra-Turrax^®^ (IKA-Werke GmbH & Co. KG, Staufen, Germany) in 2 mL of 0.9% NaCl. To this, 20 µL of radiolabeled fumonisins (ISs) and 2 mL of acetonitrile/methanol (1:1, *v*/*v*) were added. Homogenates were stirred for 2 h at room temperature and then centrifuged for 15 min at 3000× *g*. The supernatant was extracted with hexane, and the aqueous phase was applied to a FUMONIPREP^®^ column following the manufacturer’s instructions. The eluant was collected, evaporated to dryness, resuspended in 200 µL of acetonitrile/methanol (1:1, *v*/*v*), filtered, and injected into the LC-MS/MS system.

Brain homogenates were prepared by adding 1.5 mL of phosphate buffer (0.1 M, pH 7.4) to 0.5 g of brain tissue and homogenizing with an Ultra-Turrax^®^ device. Homogenates were centrifuged at 3000× *g* for 15 min, and the supernatant (S3000) was collected and stored at −80 °C until analysis.

SLs were quantified as previously described [21]. Briefly, 40 µL of S3000, 120 µL of 0.9% NaCl, and 10 μL of ISs were incubated overnight at 48 °C in 600 μL of methanol/chloroform (2:1, *v*/*v*). Hydrolysis of glycerophospholipids was performed by adding 100 µL of 1 M KOH in methanol and incubating for 2 h at 37 °C. KOH was neutralized with 10 µL of 50% acetic acid, and the homogenate was centrifuged at 4500× *g* for 15 min. The residue was re-extracted with 600 µL of methanol/chloroform (2:1), and both supernatants were pooled, evaporated to dryness, resuspended in 200 µL of methanol, filtered, and injected into the LC–MS/MS system.

OLs were measured as previously described [15]. In brief, 100 µL of S3000 was added to a hemolysis tube containing 1.800 µL of 0.9% NaCl, 160 µL of EtOH, 50 µL of 50% acetic acid, 40 µL of IS mixture, 10 µL of 12-[[(tricyclo[3.3.1.13,7]dec-1-ylamino)carbonyl]amino]-dodecanoic acid (5 mg/mL in DMSO), and 10 µL of an antioxidant cocktail (EDTA 2 mg/mL, indomethacin 2 mg/mL, BHT 0.2 mg/mL, triphenylphosphine 0.2 mg/mL in water/methanol/ethanol 2:1:1, *v*/*v*/*v*). OLs were extracted over 60 mg Oasis HLB 3 cc columns under a maximum vacuum of 20 mm Hg. Columns were washed with 2 mL of 5% MeOH and eluted with 1 mL of MeOH followed by 2 mL of ethyl acetate into a hemolysis tube containing 5 µL of glycerol/methanol (30:70, *v*/*v*). The eluate was dried at 40 °C, resuspended in 200 µL of EtOH, filtered, and injected into the LC-MS/MS system.

### 2.4. LC-MS/MS Analysis of Fumonisins, Sphingolipids and Oxylipins

Analyte separation was performed on an Agilent Poroshell 120 column (3.0 × 50 mm, 2.7 µm) using an Agilent 1260 autosampler binary pump (Santa Clara, CA, USA) at a flow rate of 0.3 mL/min, following previously described conditions for FB [22], SL [21], and OLs [15]. Detection of FB and SLs was carried out using dynamic multiple reaction monitoring (MRM) on an Agilent 6410 triple quadrupole spectrometer with positive electrospray ionization at 300 °C, gas flow of 10 L/min, and capillary voltage of 4000 V under 25 psi. OLs were analyzed under identical conditions using negative electrospray ionization. MRM parameters, retention times, and method validation for SLs and OLs are detailed in Tables S1 and S2.

Chromatograms were processed using Agilent MassHunter Quantitative Analysis software B.05.291.0 with quadratic regression and a 1/x^2^ weighting factor. The methods exhibited good linearity over a wide concentration range (Tables S3 and S4), consistent with previous reports [15,21]. LOQs for SLs and OLs corresponded to the lowest validated concentration. Precision was considered acceptable, with a relative standard deviation (RSD) of ≤20%. Intra-day repeatability, determined from IS recovery, is presented in Table S5, with RSD < 20% deemed acceptable. For SLs and OLs lacking available standards, concentrations were estimated using calibration curves of structurally similar analytes within the same class. Final brain concentrations were adjusted for recovery using the corresponding IS. The limits of detection (LOD) and quantification (LOQ) for FB1 were 0.1 and 0.5 nmol/kg, respectively.

### 2.5. Cytokines and Enzymes Activities

Cytokines (IL-6, IL-10, IL-1β, and TNFα) were measured using commercial chicken ELISA kits (Abbexa, Cambridge, UK), and enzyme activities of PLAc and COX2 were determined using commercial chicken ELISA kits (MyBiosource, San Diego, CA, USA). All procedures were carried out following the manufacturers’ instructions. Absorbance readings were performed on a VERSAmax tunable microplate reader (Molecular Devices, San Jose, CA, USA). IL-1β and TNFα were assayed without dilution and in singlicate, whereas IL-6, IL-10, PLAc, and COX2 were assayed in duplicate with a twofold dilution. CK concentrations are expressed in pg/mL, and enzyme concentrations in ng/mL.

### 2.6. Analysis Strategies and Statistics

Data are presented as mean ± SD in tables, while figures display error bars as SE for clarity. All statistical analyses were performed using XLSTAT Biomed software (version 2018.1.1 62926), Addinsoft, Bordeaux, France). Multivariate analysis was conducted using partial least squares discriminant analysis (PLS-DA). Models were considered sufficiently robust when Q^2^ exceeded 0.5. If R^2^X was low, the number of analytes was reduced to improve model quality, enhancing parsimony and interpretability by selecting analytes with the highest variable importance in projection (VIP) scores. VIP thresholds of 1.0 and 1.1 were used for SLs and OLs, respectively. Univariate analyses were performed to quantify differences between control, FB14, and FB21 groups. Normality was assessed for all analytes using the Shapiro–Wilk test. Normally distributed data were analyzed by ANOVA followed by Tukey’s post hoc test, while non-normally distributed data were analyzed using the Kruskal–Wallis test. Pearson correlation analyses were performed within each group to examine potential interactions between SLs, OLs, cytokines, and enzyme activities. Differences were considered statistically significant at *p* < 0.05, with significance indicated as * for 0.01 < *p* < 0.05, ** for 0.001 < *p* ≤ 0.01, and *** for *p* ≤ 0.001, or by different letters or color codes in the Figures.

## 3. Results

### 3.1. Fumonisins in the Brain and Effects on the Brain Sphingolipidome

**Figure 1:**
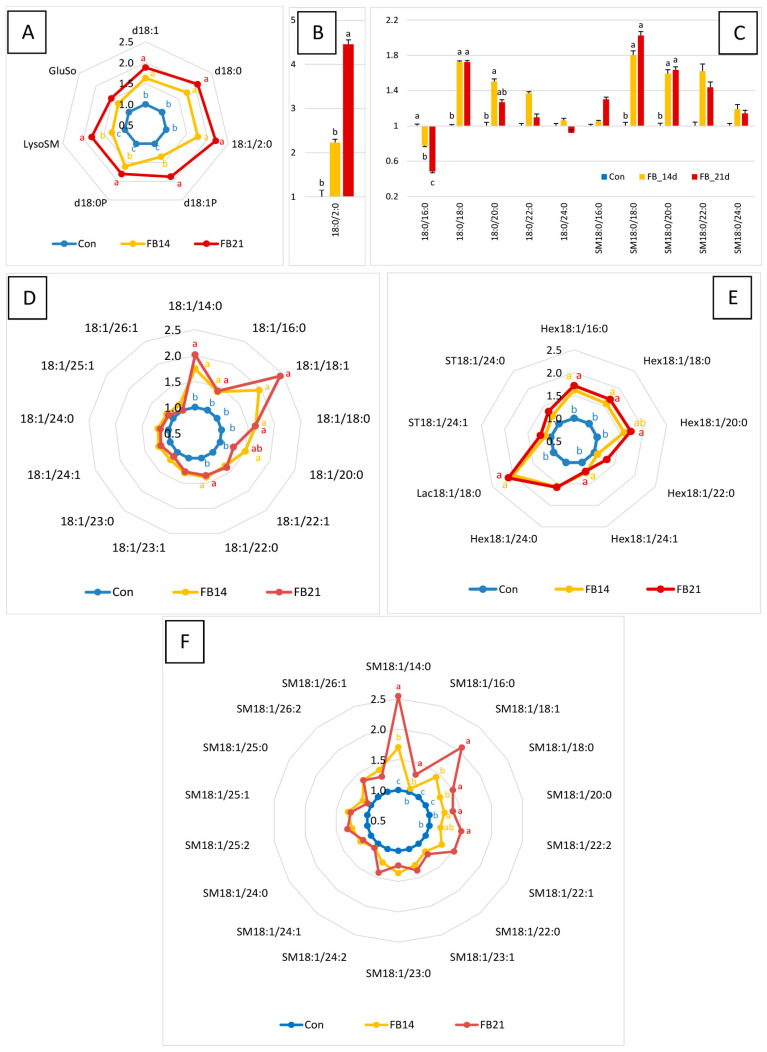
Abundance of SLs in the brains of control chickens (Con, unexposed to FB) and those fed a diet containing 14.6 mg FB1 + FB2/kg for 14 days (FB14) or 21 days (FB21). Results are expressed as mean fold change ± SE relative to unexposed controls (n = 10 per group). Differences between groups were assessed by ANOVA, with statistically significant differences (Tukey’s test, *p* < 0.05) indicated by different letters. Panels (**A**,**B**) show sphingoid bases (SBs) and their derivatives; (**C**) dihydroceramides (DHCers) and dihydrosphingomyelins (DHSMs); (**D**) ceramides (Cers); (**E**) glycosylceramides (GlyCers) including sulfatides (STs); and (**F**) sphingomyelins (SMs).

Feeding chickens with 14.6 mg FB1 + FB2 mg/kg for 14 and 21 days resulted in brain FB1 concentrations of 1 and 1.9 nmol/kg, respectively, while no FB1 was detected in control animals (LOD 0.1 nmol/kg). Concentrations of SLs showing significant changes (ANOVA, *p* < 0.05) are presented in Table 1, with complete results in Table S6. Partial least squares discriminant analysis (PLS-DA) across the three groups (Con, FB14, FB21) did not yield a robust model (Figure S1A). However, pairwise PLS-DA between Con and FB14, and Con and FB21, produced robust models with 100% specificity and sensitivity and Q^2^ values of 0.709 and 0.843, respectively (Figure S1B,C). The most discriminating SLs in these models are listed in Table 1, most of which corresponded to SLs whose concentrations changed significantly. Complementary representations of SLs as fold change relative to controls are shown in Figure 1.

In control brains, C18:0 was the most abundant Cer, representing 46.4% of total Cers, followed by C24:1 (15.2%) and C16:0 (13.6%). Other Cers included C20:0 (6.9%), C22:1 (6.0%), C22:0 (4.8%), C24:2 (2.3%), and C24:0 (1.9%), while remaining Cers were <1%. Sphingomyelin (SM) abundances were generally similar to Cer, except for SM18:1/16:0 and SM18:1/22:0, representing 3.1% and 10% of total SMs, respectively.

FB exposure caused a significant increase in nearly all SBs, their N-acetylated and phosphorylated forms, and in lysosphingolipids GluSo and LysoSM (Figure 1A,B). These increases were generally more pronounced at 21 days than at 14 days, particularly for d18:1P and d18:0P. No significant changes were observed in Sa:So or Sa1P:So1P ratios (Table S6).

Effects on DHCers were analyte-dependent: 18:0/16:0 decreased, more markedly at 21 days, whereas C18–C20 DHCers increased. 18:0/22:0 was slightly increased, and 18:0/24:0 remained unchanged. Total DHCer levels were unaffected, while the C22–24:C16 ratio increased. DHSMs largely mirrored DHCer changes, except for SM18:0/16:0, which was unchanged. Total DHSM and the C22–24:C16 DHSM ratio did not differ from controls (Figure 1C, Table S6).

C14-20 Cer increased significantly after FB exposure, with smaller increases for 18:1/16:0 and 18:1/18:0 compared to 18:1/14:0 and 18:1/18:1 (Figure 1D). C22–26 Cer were unaffected. Total Cer increased, while the C22–24:C16 Cer ratio remained unchanged (Table 1 and Table S6). SM changes generally mirrored those of Cer (Figure 1F), with smaller increases in SM18:1/16:0 and SM18:1/18:0 compared to SM18:1/14:0 and SM18:1/18:1. SM C22–26 were not significantly affected, and total SM increased without changes in the C22–24:C16 ratio (Table 1 and Table S6).

**Figure 2:**
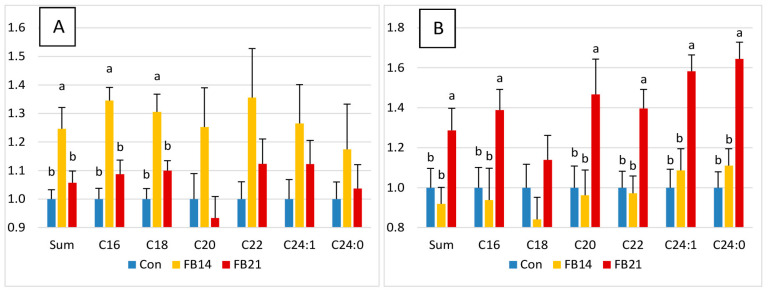
(**A**) Cer:SM ratios and (**B**) So1P:Cer ratios in the brains of chickens fed a control, mycotoxin-free diet (Con) or a diet containing 14.6 mg FB1 + FB2/kg for 14 days (FB14) or 21 days (FB21). Ratios are shown for both the total and individual sphingolipid chain lengths. Values are expressed as mean ± SE (n = 10 per group). Differences between groups were assessed by ANOVA, with statistically significant differences (Tukey’s test, *p* < 0.05) indicated by different letters.

Cer:SM and So1P:Cer ratios are presented in Figure 2. Cer:SM ratios were significantly increased at 14 days for total Cers and for C16 and C18 (Figure 2A), with trends for C20–C24 that did not reach significance due to individual variability. At 21 days, Cer:SM ratios were similar to controls (Figure 2A). The So1P:Cer ratio was unchanged at 14 days but significantly increased at 21 days for total Cers and all individual Cers except C18 (Figure 2B). FB effects on GlyCers and Cer sulfatides were variable. HexCers C16-20 and C24:1, as well as Lac18:1/18:0, increased strongly by 14 days, with no further increase at 21 days (Figure 1E). HexCer 18:1/22:0, HexCer 18:1/24:0, ST18:1/24:1, and ST18:1/24:0 were unaffected. The C22–24:C16 ratio in HexCers decreased at 14 days (Table 1).

### 3.2. Effect of FB on the Brain Oxylipidome

Brain OL concentrations are presented in Table 2. Almost all measured OLs were significantly increased after 21 days of FB exposure, whereas only PGF2α was significantly elevated at 14 days. PLS-DA across all three groups did not produce a robust model (Figure S2A) and the pairwise PLS-DA between Con and FB14 similarly failed to discriminate groups (Figure S2B). In contrast, PLS-DA between Con and FB21 yielded a robust model with 100% sensitivity and specificity and a Q^2^ of 0.695 (Figure S2C). The 22 most important variables in projection are listed in Table 2: 10 derived from AA, 10 from DHA, 1 from LA, and 1 from DPA, corresponding to 11 ω6 OLs (AA and LA) and 11 ω3 OLs (DHA and DPA).

**Figure 3:**
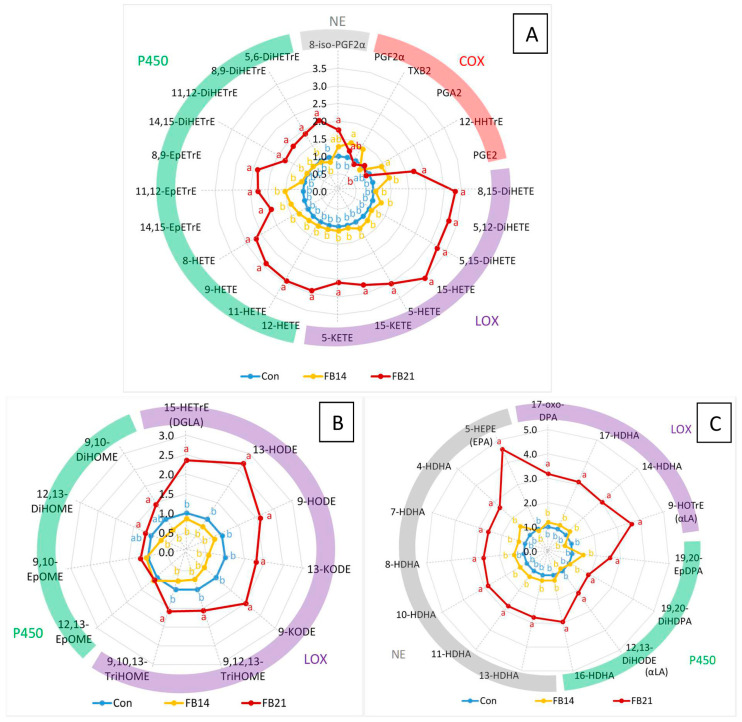
Effects of feeding a diet containing 14.6 mg FB1 + FB2/kg for 14 days (FB14) and 21 days (FB21) on OL concentrations are presented as mean fold change ± SE relative to unexposed controls (n = 10 per group) for (**A**) OLs derived from arachidonic acid (AA), (**B**) OLs from linoleic acid (LA), and (**C**) OLs from docosahexaenoic acid (DHA). Differences between groups were assessed by ANOVA, with statistically significant differences (Tukey’s test, *p* < 0.05) indicated by different letters.

The relative abundance of OLs in the control group varied depending on the PUFA and synthetic pathway. AA was the most abundant PUFA, accounting for 67.5% of total OLs, followed by LA (14.9%) and DHA (9%). OLs derived from DPA, DGLA, and αLA accounted for 2.3%, 0.9%, and 0.6%, respectively (Figure 3A). The LOX and P450 pathways contributed 42% and 37%, respectively, to the total OL abundance, followed by the non-enzymatic (NE) pathway (18%) and the COX pathway (3%).

After 14 days of FB exposure, OLs derived from AA via COX were slightly increased, except for PGA2 (Figure 3A). By 21 days, most COX-derived OLs returned to control levels, except for 8-iso-PGF2α and PGE2, which remained elevated (Figure 3A). LOX- and P450-derived OLs were unaffected at 14 days but markedly increased at 21 days, with LOX-derived OL showing the largest increases; 15-HETE exhibited the greatest elevation. OLs from LA and DHA showed variable changes (Figure 3B,C). At 14 days, most were unaffected, whereas at 21 days they were generally increased, with DHA-derived OLs showing more pronounced increases than those from LA. Among pathways, LOX-derived OLs increased more than those formed by P450 or NE.

Concerning ratios of diols to parent epoxides, only the 19,20-DiHDPA:19,20-EpDPE ratio was significantly increased at both 14 and 21 days; other ratios (9,10-DiHHOME:9,10-EpOME, 12,13-DiHHOME:12,13-EpOME, 11,12-DiHETrE:11,12-EpETrE, 8,9DiHETrE:8,9-EpETrE, or 5,6-DiHETrE:5,6-EpETrE) were unaffected. The 9-HODE:13-HODE ratio decreased significantly at 21 days. These results indicate that FB moderately increased COX-derived OLs after 14 days, which was insufficient to discriminate animals by PLS-DA. By 21 days, a marked increase occurred in most OLs, particularly those derived from LOX, with smaller increases in P450- and NE-derived OLs.

### 3.3. Effect of FB on PLA2c and COX2 Activities

**Figure 4:**
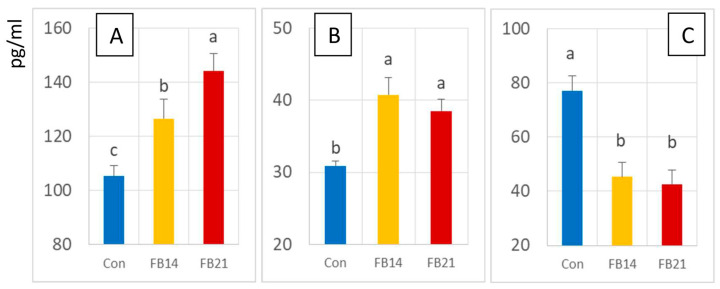
Activities of (**A**) cytosolic phospholipase A2 (PLA2c) and (**B**) cyclooxygenase-2 (COX2) and concentrations of (**C**) IL-6 in the brains of chickens fed a control, mycotoxin-free diet (Con) or a diet containing 14.6 mg FB1 + FB2/kg for 14 days (FB14) or 21 days (FB21). Values are expressed as mean ± SE (n = 10 per group). Differences between groups were assessed by ANOVA, with statistically significant differences (Tukey’s test, *p* < 0.05) indicated by different letters.

PLA2c activity was significantly increased in FB-exposed chickens at 14 days and continued to rise at 21 days (Figure 4A). COX2 activity was also significantly elevated compared to controls, but no difference was observed between 14 and 21 days of exposure (Figure 4B). Effects of FB on LOX and P450 could not be assessed due to limited sample volume.

### 3.4. Effect of FB on TNFα, IL1b, IL6, and IL10 Levels

FB exposure had no significant effect observed on brain concentrations of TNFα, IL1b, and IL10 (Figure S3). In contrast, IL6 levels were significantly decreased at both 14 and 21 days compared to controls, with no further change between the two time points (Figure 4C).

### 3.5. Correlations Between Variables

**Figure 5:**
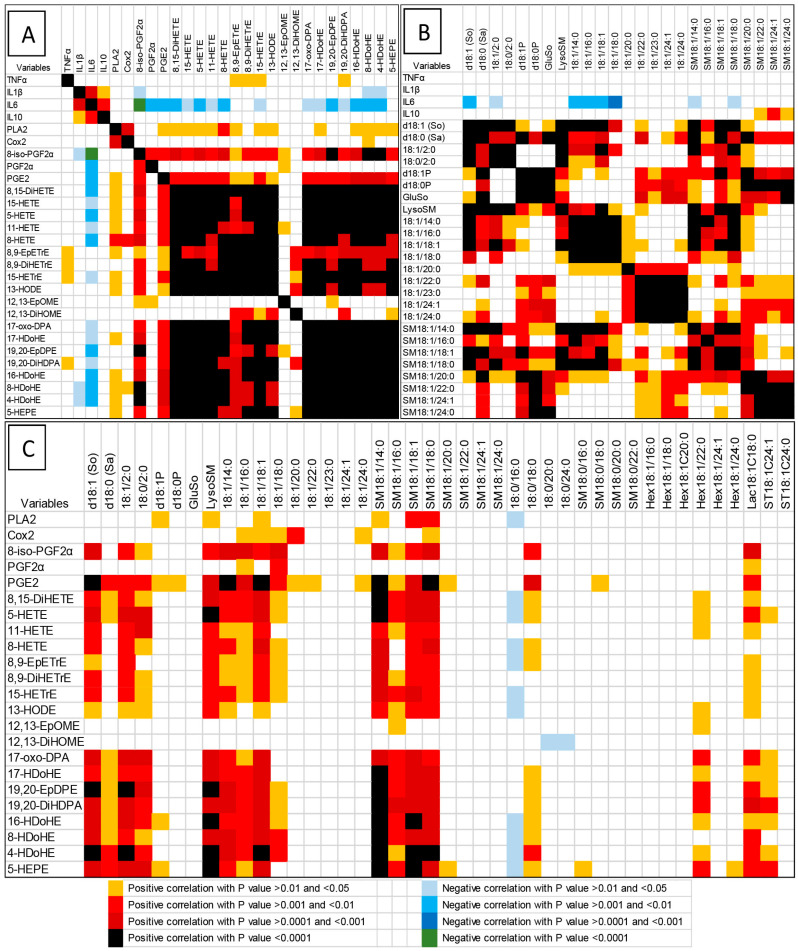
Correlation analyses between variables measured in the brains of chickens fed a control, mycotoxin-free diet (Con) or a diet containing 14.6 mg FB1 + FB2/kg for 14 days (FB14) or 21 days (FB21). Panel (**A**) shows correlations between cytokines, PLA2c and COX2 activities, and oxylipins (OLs); (**B**) between cytokines and sphingolipids (SLs); and (**C**) between PLA2c and COX2 activities, OLs, and SLs. *p* values indicate the significance of each correlation.

Potential interactions between the measured variables were investigated using correlation analyses. *p*-values for key variables are presented in Figure 5 and in Table S7.

#### 3.5.1. Correlations Between Cytokines, Oxylipins, and PLA2c and COX2 Activities

- Analysis of CK correlations revealed significant positive relationships between IL1β, IL6, and IL10, whereas TNFα was not correlated with the other CKs (Figure 5A). CKs showed no correlation with PLA2c or COX2 activities. Only weak positive correlations were observed between OLs and TNFα, while numerous negative correlations were noted between OLs and IL6, and to a lesser extent IL1β. These negative correlations were more pronounced for OLs derived from AA and DHA than from LA.
- PLA2c activity displayed positive correlations with many OLs, except for those derived from P450 or from LA (Figure 5A, Table S7). COX2 activity was strongly positively correlated with PLA2c activity but showed no correlation with any OLs, including COX-derived OLs.
- Regarding OL–OL correlations, strong and significant positive correlations were generally observed, except for PGF2α, 12,13-EpOME, and 12,13-DiHOME, as well as OLs formed by P450 epoxylases, which were weakly correlated with other OLs (Figure 5A, Table S7).

#### 3.5.2. Correlations Between Cytokines and Sphingolipids

- No significant correlations were observed between TNFα and IL1β, and SB, their derivatives, Cers, or SMs. In contrast, IL6 showed strong negative correlations with d18:1, 18:1/2:0, and C14-C18 Cers. Positive correlations were found between IL10 and C22–C24 SM (Figure 5B, Table S7).
- Strong positive correlations were observed within SL classes. SBs were positively correlated with their acetylated forms, LysoSM, and C14-C18 Cers and SMs (Figure 5B). d18:0 correlated strongly with d18:1P, d18:0P, and GluSo, which were also mutually correlated. Cer–SM correlations were size-dependent: C14-C18 Cers and SMs were strongly correlated with each other but only weakly with C20–C24 Cers and SMs, which were strongly inter-correlated (Figure 5B). DHSL and GlyCers showed strong positive correlations among analytes, with chain length playing a smaller role than observed for Cers and SMs (Figure S4, Table S7). All DHSMs were positively correlated with each other and with GlyCers, but showed no correlation with DHCers. Notably, 18:0/16:0 exhibited weak negative correlations with d18:0 and various GlyCers, distinguishing it from other analytes.

#### 3.5.3. Correlations Between PLA2c and COX2 Activities, and Oxylipins and Sphingolipids

- PLA2c activity showed significant positive correlations with d18:1P, LysoSM, 18:1/18:1, SM18:1/14:0, SM18:1/18:1, and SM18:1/18:0, and a significant negative correlation with 18:0/16:0. COX2 activity was positively correlated with 18:1/16:0, 18:1/18:1, 18:1/18:0, 18:1/20:0, 18:1/24:0, and SM18:1/18:0 (Figure 5C, Table S7).
- Except for PGF2α, 12,13-EpOME, and 12,13-DiHOME—which were generally poorly correlated with SLs—the correlations between OLs and SLs were strongly dependent on SL class and chain length (Figure 5C). Strong positive correlations were observed between OLs and d18:1, 18:1/2:0, LysoSM, and Cers and SMs in C14-C18, 18:0/18:0, Hex18:1/22:0, Lac18:1/18:0, and ST18:1/24:1. By contrast, OLs generally showed no correlation with Cers or SMs in the C20–C24 range. Correlations with DHSLs were lower than those observed for other SLs. Negative correlations were noted between OLs and 18:0/16:0 (Figure 5C).

## 4. Discussion

No significant effects of FB on the animals’ behavior, feed intake, growth, biochemical parameters, or organ weights were observed in this study, consistent with the maximum recommended FB content in poultry feed [21,23].

### 4.1. Fumonisins, Sphingolipids, and PLA2c Activity

Feeding chickens a diet containing 14.6 mg FB1 + FB2/kg for 14 and 21 days resulted in brain FB1 levels of 1.0 and 1.9 nmol/kg, respectively. This study is the first to report the presence of FB1 at low concentrations in the brain. This finding is significant because previous studies in mice have suggested that FB1 injected by three daily subcutaneous doses of 2.25 mg FB1/kg does not enter the brain unless the BBB is inflamed [7]. Although the measured concentrations of FB1 in the brain are very low—much lower than in the liver or kidneys [8,10]—they increase over time, suggesting that prolonged exposure to low doses of fumonisins is necessary for them to diffuse into the brain. This finding complements previous studies that did not detect FB1 in the brain after four or nine days of exposure to 20.8 mg of FB1 + FB2 per kg of diet [10]. Taken together, these results show that measuring FB1 or its effects on SLs in the brain requires either high doses of fumonisins or long-term exposure to low doses. This observation is consistent with previous studies that have highlighted the cumulative nature of FB1 toxicity during ELEM [1,2,4]. Additionally, the accumulation of FB1 in the brain over time aligns with previous observations of its accumulation in the livers of chickens and, to a lesser extent, pigs [24,25].

FB exposure elevated SBs and their derivatives in the brain, and increased concentrations of C14-C20 Cers and SMs, while C22–C26 species were less affected. The SL alterations were generally more pronounced than those previously reported in brain [10]. The absence of increased Sa:So, Sa1P:So1P, and C22–C24:C16 ratios, combined with elevated Cer and SM levels, indicates that the observed SL changes were not related to CerS inhibition. The absence of CerS inhibition in the brain aligns with the low FB1 concentrations detected in this tissue. FB1-mediated CerS inhibition is well-documented across species and cell types [4] and occurs in chickens’ liver and kidney at FB1 concentrations near the IC50 of 100 nM [8,26].

Elevated So1P and Sa1P concentrations at 14 and 21 days mirror increases reported in human neuroinflammatory disorders [27]. In neurons, So1P exerts protective effects against Cer-induced cytotoxicity, whereas in microglia, it promotes astrogliosis and NLRP3 inflammasome activation [28]. The increased Cer:SM ratio and the significant rise in GlyCers observed after 14 days also supports an inflammatory state, as similar elevations have been described in neuroinflammatory and neurodegenerative diseases [29,30].

This study also report for the first time that feeding FB increases in PLA2c activity at both 14 and 21 days. PLA2c activation typically depends on post-receptor signaling involving multiple phosphorylation events [31]. The positive correlations observed between PLA2c activity and So1P or LysoSM support this mechanism. So1P binding to S1P receptors may activate ERK/PKC pathways and mobilize intracellular Ca^2+^, leading to PLA2c activation [32]. LysoSM, which accumulates during inflammatory conditions [33], is generated from SMs via ceramidase activity. Ceramidases hydrolyze Cers into So, subsequently phosphorylated to So1P by SphK, thus regulating inflammation and apoptosis [34]. Acid ceramidase preferentially hydrolyzes C6:0–C18:0 Cers, neutral ceramidase acts mainly on C16–C18 Cers, and alkaline ceramidases—of which three subtypes are known—prefer unsaturated and very-long-chain Cer species [34]. The relative abundance of 18:1/18:1 compared to 18:1/16:0, 18:1/18:0, and C20–C26 Cer species observed here may therefore reflect substrate specific ceramidase activity. Interestingly, 18:1/18:1 was negatively correlated with IL6 in this study, in line with prior reports showing that its accumulation reduces proinflammatory CK production in mice [35]. The observation of elevated phospholipase A2c (PLA2c) activity in the brain is important because it has been associated with the oxidative stress and neurodegeneration that occur in neuroinflammatory and neurodegenerative diseases in humans [36]. An increase in oxidative damage was found in BALB/c mice that received 6.75 mg of FB1 per kg of body weight (BW) by subcutaneous administration for five days or 5 mg of FB1 per kg BW orally [37,38]. The same increase was found in cultured astrocyte-like C6 cells exposed to 5 µM of FB1 [39].

### 4.2. Chain Length-Dependent Effects of FB on Ceramides and Sphingomyelins

In this study, the increase in C14-C20 Cer in the brains of chickens fed FB was markedly greater than that of C22–C24 Cers, a pattern also observed for SMs. This result is difficult to compare because there is little information about the effects of FB on the brain. Increases in long-chain Cers have been reported during apoptosis in neurons and microglial cells, as well as during neurodegenerative diseases [40]. The relative abundance of Cer and SM species varies according to brain region and cell type [5,41]. Neuronal SL profiles are dominated by C18 species produced by CerS1, whereas oligodendrocytes mainly synthesize C22–C24 Cer via CerS2, which are critical components of myelin [5,42]. Astrocytes and microglial cells contain C16, C18, C20, and C24 Cer, though C18 Cer is generally less abundant in microglial cells than in astrocytes [5,41]. Therefore, the strong increase in C14–C20 Cer and SM relative to C22–C24 species in this study likely reflects a FB effect on astrocytes or microglial cells. This observation aligns with in vitro studies that have shown that glial cells are more susceptible to FB1 toxicity than neurons [20,43,44]. Notably, 15 µM FB1 disrupts lipid homeostasis and promotes oxidative stress, effects mitigated by magnolol via modulation of the PI3K/Akt pathway [6,39]. In this context, the increase in So1P observed in this study is particularly relevant, as So1P is known to activate the PI3K/Akt pathway and autophagy [45].

Taken together, these results suggest that disturbances in SL synthesis occurring in cell populations more sensitive or more exposed to FB could initiate neuroinflammation, potentially masking the direct inhibitory effects of FB on (dh)CerS.

### 4.3. Oxylipins, COX2 Activity, and Correlations with Sphingolipids

The effects of FB on OLs varied depending on the pathway responsible for its synthesis and the duration of exposure to FB. At 14 days, a moderate increase was observed in OLs derived from the COX pathway, with a significant effect only on PGF2α. At 21 days, levels of both 8-iso-PGF2α and PGE2 increased. This observation strengthens previous studies in chickens that reported a mild increase in COX-derived OLs after 9 days of exposure [15]. PGF2α is a potent cerebral vasoconstrictor, while PGE2 promotes vasodilation [13,46,47]. Elevated PGE2 concentrations have been documented in neurodegenerative and neuroinflammatory diseases [11,29]. Two COX isoforms are responsible for the production of cyclized OLs: the constitutive COX1 and the inducible COX2. The slight increase in COX2 activity observed at 14 and 21 days, indicates a weak effect of FB on COX2 enzymatic function in this study. The sustained increase in PGE2 at 21 days is consistent with previous reports showing that PGE2 is abundantly synthesized by macrophages and microglial cells during the resolution phase of inflammation under the influence of So1P [11,48].

No significant differences in OLs produced via the LOX, P450, or NE pathways were observed at 14 days, whereas nearly all increased significantly after 21 days of exposure. The increase affected OLs derived from both AA and DHA, and was more pronounced for LOX-derived products with a smaller effect on LA-derived OLs produced by P450 epoxylases. Most LOX-derived OLs, particularly those from DHA, are recognized as pro-resolving mediators of inflammation [13,46,47]. This is notably the case for 17-HDHA, whose precursor 17-HpDHA gives rise to D-series resolvins and protectins/neuroprotectins, and for 14-HDHA, derived from 14-HpDHA, which leads to maresins [49]. The sharp increases in 14- and 17-HDHA observed in this study are consistent with findings in neuroinflammatory and neurodegenerative conditions [29]. The only EPA-derived OL detected in brain, 5-HEPE (produced by 5-LOX), is known for its antioxidant role [13,46,47]. Similarly, the significant decrease in the 9-HODE:13-HODE ratio observed at 21 days reflects a shift toward anti-inflammatory signaling, as 9-HODE is pro-inflammatory whereas 13-HODE exerts opposite effects [13,46].

Correlations between OLs and SLs varied depending on both the PUFA precursor and the SL class. Correlations between OL derived from LA or αLA and SL were generally weaker than those from AA and DHA. This result can be explained by the relative concentrations of PUFAs in the brains of chickens, as well as the lower affinity of LA and αLA compared to AA and DHA for LOX and P450 enzymes [50]. Strong positive correlations were observed between OLs and Cer or SM species in the C14–C18 range, whereas no correlations were found for C20–C24 species, indicating that OL production was closely associated with increases in short- and medium-chain SLs in the brain. Altogether, the OL profile observed at 21 days indicates enhanced biosynthesis of OLs involved in the resolution of inflammation. OL synthesis in the brain is primarily attributed to microglial cells, and to a less extent astrocytes, which serve as the brain’s resident macrophages [41,48,51]. The strong correlations between C14-C18 SL and OL support a key role of Cers in modulating OLs production. The generation of proinflammatory COX-derived OL may be limited by elevated intracellular 18:1/2:0 that is known to acetylate COX2 leading to the formation of ACOX2—a variant with altered catalytic properties [52]. This hypothesis is supported by the marked increase in 15-HETE, the most elevated AA-derived OL observed, which can be produced by both 15-LOX and ACOX2 [52,53]. Additionally, 18:1/2:0 has been shown to destabilize lipid membranes and to form membrane channels [54], further supporting changes in membrane permeability, which could lead to greater diffusion of FB1 into the brain, as previously discussed.

Taken together, these results suggest that disturbances in SL synthesis, such as increased So1P or LysoSM, as well as changes in the Cer abundance, could account for the increase in PLA2c activity observed at 14 and 21 days leading to an increase in OL production.

### 4.4. Cytokines

No increase in CK concentrations was observed in the brains of chickens in this study. This result is consistent with previous work in mice [7]. Pro-inflammatory CKs such as TNFα, IL1β, and IL6 are primarily produced during the initiation of inflammation, whereas IL10 acts during its resolution [17]. Beyond their immunological functions, CKs also play crucial metabolic roles: TNFα promotes insulin resistance and inhibits ketogenesis; IL1β enhances insulin secretion and energy metabolism; and IL6 facilitates the redistribution of energy from storage tissues to energy-consuming tissues [18].

The decrease in IL6 concentrations observed in animals exposed to FB for 14 and 21 days, together with its strong negative correlation with OLs and SLs—whose concentrations were elevated by FB exposure—may reflect increased lipolytic activity resulting from enhanced PLA2c activity [18].

CK production in the brain occurs mainly in microglia and, and to a lesser extent, in astrocytes, whereas oligodendrocytes and neurons contribute minimally to IL synthesis [51,55,56]. Elevated cytokine levels are typically associated with severe neuroinflammatory conditions, such as Alzheimer’s disease [16].

Therefore, the absence of a significant increase in CKs in this study suggests that CKs did not play a major role in the variation in brain SLs and OLs measured in chickens fed FB, even if the effects of FB on other cytokines or chemokines not measured in this study cannot be completely ruled out. This pattern is consistent with a mild neuroinflammatory process, likely driven by moderate activation of astrocytes or microglial cells, effectively controlled by the compensatory production of anti-inflammatory mediators such as ω3 FA-derived OLs. A better characterization of the process and confirmation of the moderate nature of the neuroinflammation observed in this study would be possible by measuring the expression and morphological changes in the microglial marker Iba1 and the astrocytic marker GFAP.

## 5. Conclusions

Feeding chickens a diet containing 14.6 mg FB1 + FB2 for 14 and 21 days induced marked alterations in SL metabolism that cannot be attributed to CerS inhibition. These changes were characterized by an accumulation of SBs and their acetylated and phosphorylated derivatives, as well as an increase in Cers and SMs with C14-C20 chains, and in various GlyCers. The Cer:SM ratio rose after 14 days but returned to baseline by day 21, while the So1P:Cer ratio increased at 14 days and continued to rise at 21 days. Such variations are consistent with the observed increase in PLA2c activity at both time points.

Targeted OL profiling revealed a modest increase in pro-inflammatory OLs derived via COX from AA at 14 days, followed by a pronounced rise at 21 days in anti-inflammatory OLs synthesized through COX and LOX pathways from AA, as well as in OLs derived from DHA. Strong correlations between C14-C18 SL and OLs involved in inflammation resolution further support a coordinated lipid response. Altogether, these results suggest that damage to microglial cells and astrocytes may underlie the increased diffusion of FB1 into the brain. Conversely, CKs did not appear to play a major role in the inflammatory response to FB exposure.

The results of this study demonstrate that a “safe” dietary dose of FB induces measurable changes in the brain sphingolipidome and oxylipidome of chickens that correspond to neuroinflammation. Further research is needed to identify the initial molecular targets of FB and determine the consequences of long-term exposure to this food contaminant. These findings raise concerns about the potential consequences of chronic, low-level exposure to FB, particularly with regard to neuroinflammatory and neurodegenerative conditions, as direct extrapolation of the results obtained in chickens in this study cannot be applied to humans.

## Figures and Tables

**Table 1:** Sphingolipid (SL) concentrations and SL ratios in the brains of chickens fed a diet containing 14.6 mg FB1 + FB2/kg for 14 days (FB14) or 21 days (FB21) that differed significantly from those in chickens fed a mycotoxin-free control diet (Con).

Analyte	Con	FB14	FB21
d18:1 (So)	1624 ± 360 b	2644 ± 464 a	3057 ± 1019 a
d18:0 (Sa)	497 ± 144 b	869 ± 246 a	1030 ± 295 a
18:1/2:0	10.4 ± 3.6 b	18.4 ± 5.1 a	22.9 ± 10.6 a
18:0/2:0	1.7 ± 2.9 b	3.8 ± 3.1 ab	7.6 ± 7.7 a
d18:1P	192 ± 57 c	258 ± 78 b	360 ± 74 a
d18:0P	247 ± 105 c	396 ± 242 b	445 ± 163 a
LysoSM	41.7 ± 7.1 c	54.7 ± 10.9 b	75.2 ± 20 a
18:1/14:0	47.3 ± 12.3 b	82.5 ± 13.4 a	95.3 ± 32.6 a
18:1/16:0	6096 ± 1660 b	8541 ± 996 a	8629 ± 2772 a
18:1/18:1	241 ± 70 b	472 ± 123 a	587 ± 193 a
18:1/18:0	20,787 ± 3846 b	34,126 ± 6036 a	33,982 ± 5949 a
18:1/20:0	3088 ± 997 b	4641 ± 1314 a	3918 ± 940 ab
18:1/22:0	2147 ± 699 b	2966 ± 755 a	2888 ± 846 a
Sum Cer	44,758 ± 7494 b	66,379 ± 8189 a	65,295 ± 11,001 a
18:0/16:0	609 ± 120 a	474 ± 44 b	295 ± 47 c
18:0/18:0	177 ± 30 b	305 ± 36 a	305 ± 51 a
18:0/20:0	693 ± 327 b	1103 ± 272 a	906 ± 149 ab
C22–24:C16 DHCer	3.1 ± 1 b	5 ± 1.3 ab	6.8 ± 2.6 a
SM18:1/14:0	183 ± 50 c	311 ± 60 b	465 ± 171 a
SM18:1/16:0	8149 ± 1479 b	8584 ± 750 b	10,605 ± 2814 a
SM18:1/18:1	1030 ± 144 c	1474 ± 220 b	2129 ± 607 a
SM18:1/18:0	157,235 ± 13,780 c	198,668 ± 18,786 b	235,797 ± 44,454 a
SM18:1/20:0	13,642 ± 2265 b	16,963 ± 3082 a	18,802 ± 2982 a
SM18:1/22:2	231 ± 72 b	272 ± 95 ab	349 ± 105 a
Sum SM	259,738 ± 29,527 b	316,961 ± 53,456 a	361,520 ± 62,055 a
SM18:0/18:0	15,466 ± 6456 b	27,890 ± 13,213 a	31,356 ± 13,551 a
SM18:0/20:0	3587 ± 1146 b	5712 ± 2546 a	5863 ± 2030 a
Hex18:1/16:0	1108 ± 308 b	1792 ± 564 a	1901 ± 439 a
Hex18:1/18:0	111,806 ± 41,211 b	176,074 ± 76,892 a	189,296 ± 57,821 a
Hex18:1/20:0	16,124 ± 7277 b	25,580 ± 13,109 ab	27,846 ± 9877 a
Hex18:1/24:1	57,091 ± 7495 b	70,518 ± 17,187 a	68,943 ± 9740 a
C22–24:C16 HexCer	205 ± 43 a	155 ± 40 b	161 ± 48 a
Lac18:1/18:0	4011 ± 1566 b	8020 ± 3681 a	8471 ± 3545 a

Results are expressed as mean ± SD in pmol/g of brain (n = 10). Differences between groups were assessed by ANOVA, with statistically significant differences identified by Tukey’s test (*p* < 0.05) and indicated by different letters. Underlined values correspond to variables with VIP > 1 in the PLS-DA analysis shown in Figure S1.

**Table 2:** Oxylipin concentrations in the brains of chickens fed a mycotoxin-free control diet (Con) or a diet containing 14.6 mg FB1 + FB2/kg for 14 days (FB14) or 21 days (FB21).

Analyte	Fatty Acid	Enzyme	Con	FB14	FB21
8-iso-PGF2α	AA;20;6	NE	15.7 ± 8.8 b	20 ± 7.6 ab	27.5 ± 12.5 a
PGF2α	AA;20;6	COX	110 ± 30 b	156 ± 31 a	130 ± 42 ab
TXB2	AA;20;6	COX	26.8 ± 15.4	37 ± 16.1	23.8 ± 7.1
PGA2	AA;20;6	COX	47.4 ± 24.8	41 ± 16.3	49.1 ± 24.6
12-HHTrE	AA;20;6	COX	104 ± 45 ab	146 ± 62 a	93 ± 24 b
PGE2	AA;20;6	COX	15 ± 6 b	22.3 ± 11.3 b	33 ± 10.5 a
8,15-DiHETE	AA;20;6	15LOX_m	55.5 ± 40.8 b	58.6 ± 32.1 b	184 ± 99 a
5,12-DiHETE	AA;20;6	LOX_m	2.7 ± 2.1 b	3.3 ± 2.1 b	8.6 ± 3.7 a
5,15-DiHETE	AA;20;6	15LOX_m	38.9 ± 25.9 b	42 ± 25.7 b	126 ± 59 a
15-HETE	AA;20;6	15LOX/P450	3070 ± 2252 b	3597 ± 1551 b	10,713 ± 6412 a
5-HETE	AA;20;6	5LOX/P450	609 ± 389 b	742 ± 294 b	1843 ± 1059 a
15-KETE	AA;20;6	15LOX	146 ± 93 b	158 ± 66 b	402 ± 195 a
5-KETE	AA;20;6	5LOX	114 ± 67 b	128 ± 47 b	295 ± 133 a
12-HETE	AA;20;6	LOX/P450	482 ± 281 b	540 ± 257 b	1407 ± 713 a
11-HETE	AA;20;6	P450/NE	1728 ± 1161 b	1967 ± 735 b	5077 ± 2927 a
9-HETE	AA;20;6	P450	357 ± 230 b	418 ± 272 b	1035 ± 488 a
8-HETE	AA;20;6	P450	117 ± 63 b	150 ± 76 b	316 ± 144 a
14,15-EpETrE	AA;20;6	P450	8.5 ± 3.6 b	11.9 ± 7.6 b	16.8 ± 5.7 a
11,12-EpETrE	AA;20;6	P450	1.4 ± 0.2 b	2.1 ± 1.1 b	3.1 ± 0.8 a
8,9-EpETrE	AA;20;6	P450	173 ± 142 b	187 ± 97 b	411 ± 218 a
14,15-DiHETrE	AA;20;6	P450	22.6 ± 11.4 b	23.1 ± 5.6 b	39.6 ± 14.4 a
11,12-DiHETrE	AA;20;6	P450	27.3 ± 15.3 b	27.4 ± 6.7 b	49.5 ± 17.7 a
8,9-DiHETrE	AA;20;6	P450	51.1 ± 31.6 b	48.7 ± 15.1 b	96.5 ± 36.5 a
5,6-DiHETrE	AA;20;6	P450	59.2 ± 34.9 b	50.9 ± 19.2 b	123.7 ± 51.7 a
15-HETrE	DGLA;20;6	15LOX_m	99 ± 53.5 b	84.7 ± 34.2 b	233 ± 115 a
13-HODE	LA;18;6	15LOX	279 ± 171 b	216 ± 73 b	754 ± 421 a
9-HODE	LA;18;6	LOX	79.4 ± 46.3 b	62.8 ± 23 b	166 ± 65 a
13-KODE	LA;18;6	15LOX_m	65.6 ± 62.1 b	37.4 ± 17.5 b	119 ± 56 a
9-KODE	LA;18;6	LOX_m	961 ± 943 b	585 ± 242 b	1931 ± 1043 a
9,12,13-TriHOME	LA;18;6	15LOX	80.2 ± 54.2 b	58.6 ± 12.6 b	125 ± 38 a
9,10,13-TriHOME	LA;18;6	LOX	71.1 ± 45.7 b	55 ± 10.5 b	113 ± 34 a
12,13-EpOME	LA;18;6	P450	37.8 ± 42.9	42.2 ± 46.2	41.4 ± 41.7
9,10-EpOME	LA;18;6	P450	36.6 ± 28.4	38.2 ± 32.5	43.6 ± 30.4
12,13-DiHOME	LA;18;6	P450_m	11.9 ± 6.5 ab	8.6 ± 2.2 b	13.7 ± 3.7 a
9,10-DiHOME	LA;18;6	P450_m	11.5 ± 7.7 ab	7.7 ± 2.3 b	16.6 ± 6.2 a
17-oxo-DPA	DPA;22;3	LOX	255 ± 144 b	303 ± 123 b	810 ± 443 a
17-HDHA	DHA;22;3	15LOX/NE	104 ± 64 b	122 ± 69 b	323 ± 146 a
14-HDHA	DHA;22;3	15LOX/NE	415 ± 256 b	504 ± 255 b	1244 ± 617 a
19,20-EpDPA	DHA;22;3	P450	139 ± 74 b	203 ± 58 b	357 ± 150 a
19,20-DiHDPA	DHA;22;3	P450	9.9 ± 4.8 b	10.5 ± 3 b	19.1 ± 8.1 a
16-HDHA	DHA;22;3	NE	119 ± 67 b	145 ± 59 b	352 ± 156 a
13-HDHA	DHA;22;3	NE	117 ± 51 b	144 ± 51 b	325 ± 146 a
11-HDHA	DHA;22;3	NE	107 ± 50 b	137 ± 71 b	296 ± 103 a
10-HDHA	DHA;22;3	NE	66.3 ± 38.7 b	87.1 ± 38.6 b	188 ± 83 a
8-HDHA	DHA;22;3	NE	154 ± 75 b	216 ± 95 b	410 ± 164 a
7-HDHA	DHA;22;3	NE	36.6 ± 20.4 b	46.5 ± 14.5 b	94.2 ± 25 a
4-HDHA	DHA;22;3	NE	234 ± 137 b	357 ± 139 b	625 ± 217 a
5-HEPE (EPA)	EPA;20;3	NE	2.8 ± 2.6 b	2.6 ± 2.2 b	12.8 ± 8.5 a
9-HOTrE	αLA;18;3	5LOX	1.8 ± 1.6 b	1.3 ± 1.1 b	6.5 ± 3.8 a
12,13-DiHODE	αLA;18;3	P450	60.6 ± 52.2 b	53.4 ± 9.7 b	128 ± 40 a

Results are expressed as mean ± SD in ng/g of brain (n = 10). Differences between groups were assessed by ANOVA, with statistically significant differences identified by Tukey’s test (*p* < 0.05) and indicated by different letters. Underlined values correspond to variables with VIP > 1.1 in the PLS-DA analysis shown in Figure S2.

## Data Availability

The raw data supporting the conclusions of this article will be made available by the authors on request.

## Supplementary Materials

The following supporting information can be downloaded at: https://www.mdpi.com/article/10.3390/antiox15050546/s1, Table S1. MRM parameters and retention times of sphingolipids quantified in this study; Table S2. MRM parameters and retention times of oxylipins quantified in this study; Table S3. Linearity of the method measured for the sphingolipids available as standards; Table S4. Linearity of the method measured for the oxylipins available as standards; Table S5. Intra-day recovery of the internal standards of sphingolipids and oxylipins measured in the brain of chickens; Table S6. Effect of 14 and 21 days of fumonisins exposure on the sphingolipids measured in the brain of chickens; Table S7. Sphingolipids, oxylipins, cytokines and enzyme activities Pearson *p*-value; Figure S1. PLS-DA analysis of the brain sphingolipid profile; Figure S2. PLS-DA analysis of the brain oxylipin profile; Figure S3. Concentrations of TNFα, IL-1β, and IL-10 in the brains; Figure S4. Correlation analyses between dihydrosphingolipids and glycosylceramides in the brains.

## Abbreviations

The following abbreviations are used in this manuscript:

AA	Arachidonic acid
BBB	Blood–brain barrier
Cer	Ceramide
CerS	Ceramide synthase
CK	Cytokines
COX2	Cyclooxygenase 2
DGLA	Di-homo-gamma-linolenic acid
DHA	Docosahexaenoic
DHCer	Dihydroceramide
DHSL	Dihydrosphingolipid
DHSM	Dihydrosphingomyelin
DPA	Docosapentoenoic acid
EPA	Eicosapentaenoic
FA	Fatty acid
FB	Fumonisin B
HexCer	Monohexosylceramide
HPLC	High-performance liquid chromatography
GlyCer	Glucosylceramide
IS	Internal standard
LA	Linolenic acid
LacCer	Lactosylceramide
LC-MS/MS	Liquid Chromatography coupled to tandem Mass Spectrometry
Lox	Lipoxygenase
NE	Non-enzymatic
OL	Oxylipin
PLA2c	Phospholipase A2c
PLS-DA	Partial least squares discriminant analysis
PUFA	Polyunsaturated fatty acid
SL	Sphingolipid
Sa	Sphinganine
Sa1P	Sphinganine 1-phosphate
SB	Sphingoid base
SM	Sphingomyelin
So	Sphingosine
So1P	Sphingosine 1-phosphate
SphK	Sphingosine kinase
VIP ST	Variable important in the projection Sulfatides

## References

[1] Anumudu C.K., Ekwueme C.T., Uhegwu C.C., Ejileugha C., Augustine J., Okolo C.A., Onyeaka H. (2025). A Review of the Mycotoxin Family of Fumonisins, Their Biosynthesis, Metabolism, Methods of Detection and Effects on Humans and Animals. Int. J. Mol. Sci..

[2] Gao Z., Luo K., Zhu Q., Peng J., Liu C., Wang X., Li S., Zhang H. (2023). The Natural Occurrence, Toxicity Mechanisms and Management Strategies of Fumonisin B1: A Review. Environ. Pollut..

[3] Niaz W., Iqbal S.Z., Ahmad K., Majid A., Haider W., Li X. (2025). Mycotoxins: A Comprehensive Review of Its Global Trends in Major Cereals, Advancements in Chromatographic Detections and Future Prospectives. Food Chem. X.

[4] Riley R.T., Merrill A.H. (2019). Ceramide Synthase Inhibition by Fumonisins: A Perfect Storm of Perturbed Sphingolipid Metabolism, Signaling, and Disease. J. Lipid Res..

[5] McInnis J.J., Sood D., Guo L., Dufault M.R., Garcia M., Passaro R., Gao G., Zhang B., Dodge J.C. (2024). Unravelling Neuronal and Glial Differences in Ceramide Composition, Synthesis, and Sensitivity to Toxicity. Commun. Biol..

[6] Wang Y., Cheng D., He J., Liu S., Wang X., Wang M. (2025). Magnolol Protects C6 Glioma Cells against Neurotoxicity of FB1 via Modulating PI3K/Akt and Mitochondria-Associated Apoptosis Signaling Pathways. Environ. Pollut..

[7] Osuchowski M.F., He Q., Sharma R.P. (2005). Endotoxin Exposure Alters Brain and Liver Effects of Fumonisin B1 in BALB/c Mice: Implication of Blood Brain Barrier. Food Chem. Toxicol..

[8] Guerre P., Lassallette E., Beaujardin-Daurian U., Travel A. (2024). Fumonisins Alone or Mixed with Other Fusariotoxins Increase the C22-24:C16 Sphingolipid Ratios in Chicken Livers, While Deoxynivalenol and Zearalenone Have No Effect. Chem. Biol. Interact..

[9] Ayub M., Jin H.-K., Bae J.-S. (2021). Novelty of Sphingolipids in the Central Nervous System Physiology and Disease: Focusing on the Sphingolipid Hypothesis of Neuroinflammation and Neurodegeneration. Int. J. Mol. Sci..

[10] Guerre P., Matard-Mann M., Nyvall Collén P. (2022). Targeted Sphingolipid Analysis in Chickens Suggests Different Mechanisms of Fumonisin Toxicity in Kidney, Lung, and Brain. Food Chem. Toxicol..

[11] Sheremeta C.-L., Yarlagadda S., Smythe M.L., Noakes P.G. (2024). Prostaglandins in the Inflamed Central Nervous System: Potential Therapeutic Targets. Curr. Drug Targets.

[12] Sun G.Y., Geng X., Teng T., Yang B., Appenteng M.K., Greenlief C.M., Lee J.C. (2021). Dynamic Role of Phospholipases A2 in Health and Diseases in the Central Nervous System. Cells.

[13] Gabbs M., Leng S., Devassy J.G., Monirujjaman M., Aukema H.M. (2015). Advances in Our Understanding of Oxylipins Derived from Dietary PUFAs. Adv. Nutr..

[14] Sambra V., Echeverria F., Valenzuela A., Chouinard-Watkins R., Valenzuela R. (2021). Docosahexaenoic and Arachidonic Acids as Neuroprotective Nutrients throughout the Life Cycle. Nutrients.

[15] Guerre P., Lassallette E., Guerre A., Tardieu D. (2024). Effects of the Maximum Recommended Levels of Fumonisins in the EU on Oxylipin Profiles in the Liver and Brain of Chickens. Antioxidants.

[16] Godoy J.B., Vialle R.A., Dos Santos L., Raittz R.T., Wang Y., Menon V., De Jager P.L., Schneider J.A., Tasaki S., Bennett D.A. (2025). Cytokine Expression Profile in the Human Brain of Older Adults. J. Neuroinflamm..

[17] Griffith J.W., Sokol C.L., Luster A.D. (2014). Chemokines and Chemokine Receptors: Positioning Cells for Host Defense and Immunity. Annu. Rev. Immunol..

[18] de Baat A., Trinh B., Ellingsgaard H., Donath M.Y. (2023). Physiological Role of Cytokines in the Regulation of Mammalian Metabolism. Trends Immunol..

[19] Osuchowski M.F., Edwards G.L., Sharma R.P. (2005). Fumonisin B1-Induced Neurodegeneration in Mice after Intracerebroventricular Infusion Is Concurrent with Disruption of Sphingolipid Metabolism and Activation of Proinflammatory Signaling. Neurotoxicology.

[20] Osuchowski M.F., Sharma R.P. (2005). Fumonisin B1 Induces Necrotic Cell Death in BV-2 Cells and Murine Cultured Astrocytes and Is Antiproliferative in BV-2 Cells While N2A Cells and Primary Cortical Neurons Are Resistant. Neurotoxicology.

[21] Lassallette E., Collén P.N., Guerre P. (2023). Targeted Sphingolipidomics Indicates Increased C22-C24:16 Ratios of Virtually All Assayed Classes in Liver, Kidney, and Plasma of Fumonisin-Fed Chickens. Ecotoxicol. Environ. Saf..

[22] Tardieu D., Travel A., Metayer J.-P., Le Bourhis C., Guerre P. (2019). Fumonisin B1, B2 and B3 in Muscle and Liver of Broiler Chickens and Turkey Poults Fed with Diets Containing Fusariotoxins at the EU Maximum Tolerable Level. Toxins.

[23] Schrenk D., Bignami M., Bodin L., Chipman J.K., Del Mazo J., Grasl-Kraupp B., Hogstrand C., Leblanc J.-C., Nielsen E., EFSA Panel on Contaminants in the Food Chain (CONTAM) (2022). Assessment of Information as Regards the Toxicity of Fumonisins for Pigs, Poultry and Horses. EFSA J..

[24] Laurain J., Tardieu D., Matard-Mann M., Rodriguez M.A., Guerre P. (2021). Fumonisin B1 Accumulates in Chicken Tissues over Time and This Accumulation Was Reduced by Feeding Algo-Clay. Toxins.

[25] Lassallette E., Pierron A., Tardieu D., Reymondaud S., Gallissot M., Rodriguez M.A., Collén P.N., Roy O., Guerre P. (2025). Biomarkers of Fumonisin Exposure in Pigs Fed the Maximum Recommended Level in Europe. Toxins.

[26] Wang E., Norred W.P., Bacon C.W., Riley R.T., Merrill A.H. (1991). Inhibition of Sphingolipid Biosynthesis by Fumonisins. Implications for Diseases Associated with Fusarium Moniliforme. J. Biol. Chem..

[27] Grassi S., Mauri L., Prioni S., Cabitta L., Sonnino S., Prinetti A., Giussani P. (2019). Sphingosine 1-Phosphate Receptors and Metabolic Enzymes as Druggable Targets for Brain Diseases. Front. Pharmacol..

[28] Alam S., Afsar S.Y., Wolter M.A., Volk L.M., Mitroi D.N., Meyer Zu Heringdorf D., van Echten-Deckert G. (2023). S1P Lyase Deficiency in the Brain Promotes Astrogliosis and NLRP3 Inflammasome Activation via Purinergic Signaling. Cells.

[29] Chiurchiù V., Tiberi M., Matteocci A., Fazio F., Siffeti H., Saracini S., Mercuri N.B., Sancesario G. (2022). Lipidomics of Bioactive Lipids in Alzheimer’s and Parkinson’s Diseases: Where Are We?. Int. J. Mol. Sci..

[30] Huh Y.E., Park H., Chiang M.S.R., Tuncali I., Liu G., Locascio J.J., Shirvan J., Hutten S.J., Rotunno M.S., Viel C. (2021). Glucosylceramide in Cerebrospinal Fluid of Patients with GBA-Associated and Idiopathic Parkinson’s Disease Enrolled in PPMI. npj Park. Dis..

[31] Zhang H.-J., Chen Y.-T., Hu X.-L., Cai W.-T., Wang X.-Y., Ni W.-F., Zhou K.-L. (2023). Functions and Mechanisms of Cytosolic Phospholipase A2 in Central Nervous System Trauma. Neural Regen. Res..

[32] Gomez-Larrauri A., Larrea A., Martín C., Gomez-Muñoz A. (2025). The Critical Roles of Bioactive Sphingolipids in Inflammation. J. Biol. Chem..

[33] Ge D., Yue H.-W., Liu H.-H., Zhao J. (2018). Emerging Roles of Sphingosylphosphorylcholine in Modulating Cardiovascular Functions and Diseases. Acta Pharmacol. Sin..

[34] Duarte C., Akkaoui J., Yamada C., Ho A., Mao C., Movila A. (2020). Elusive Roles of the Different Ceramidases in Human Health, Pathophysiology, and Tissue Regeneration. Cells.

[35] Wang K., Xu R., Snider A.J., Schrandt J., Li Y., Bialkowska A.B., Li M., Zhou J., Hannun Y.A., Obeid L.M. (2016). Alkaline Ceramidase 3 Deficiency Aggravates Colitis and Colitis-Associated Tumorigenesis in Mice by Hyperactivating the Innate Immune System. Cell Death Dis..

[36] Hugo C., Asante I., Sadybekov A., Katritch V., Yassine H.N. (2024). Development of Calcium-Dependent Phospholipase A2 Inhibitors to Target Cellular Senescence and Oxidative Stress in Neurodegenerative Diseases. Antioxid. Redox Signal..

[37] Krishnaswamy K., Manasa V., Khan M.T., Serva Peddha M. (2024). Apocynin Exerts Neuroprotective Effects in Fumonisin B1-Induced Neurotoxicity via Attenuation of Oxidative Stress and Apoptosis in an Animal Model. J. Food Sci..

[38] Yang D., Ye Y., Huang Y., Huang H., Sun J., Wang J.-S., Tang L., Gao Y., Sun X. (2023). Effects of FB1 and HFB1 on Autonomous Exploratory and Spatial Memory and Learning Abilities in Mice. J. Agric. Food Chem..

[39] Wang X., Cheng D., Liu L., Yu H., Wang M. (2024). Magnolol Ameliorates Fumonisin B1-Induced Oxidative Damage and Lipid Metabolism Dysfunction in Astrocyte-like C6 Cells. Chemosphere.

[40] Choudhary P., Kumari S., Bagri K., Deshmukh R. (2025). Ceramide: A Central Regulator in Alzheimer’s Disease Pathogenesis. Inflammopharmacology.

[41] Fitzner D., Bader J.M., Penkert H., Bergner C.G., Su M., Weil M.-T., Surma M.A., Mann M., Klose C., Simons M. (2020). Cell-Type- and Brain-Region-Resolved Mouse Brain Lipidome. Cell Rep..

[42] Ozgen H., Baron W., Hoekstra D., Kahya N. (2016). Oligodendroglial Membrane Dynamics in Relation to Myelin Biogenesis. Cell. Mol. Life Sci..

[43] Stockmann-Juvala H., Naarala J., Loikkanen J., Vähäkangas K., Savolainen K. (2006). Fumonisin B1-Induced Apoptosis in Neuroblastoma, Glioblastoma and Hypothalamic Cell Lines. Toxicology.

[44] Stockmann-Juvala H., Mikkola J., Naarala J., Loikkanen J., Elovaara E., Savolainen K. (2004). Oxidative Stress Induced by Fumonisin B1 in Continuous Human and Rodent Neural Cell Cultures. Free Radic. Res..

[45] Xiao S., Peng K., Li C., Long Y., Yu Q. (2023). The Role of Sphingosine-1-Phosphate in Autophagy and Related Disorders. Cell Death Discov..

[46] Shinto L.H., Raber J., Mishra A., Roese N., Silbert L.C. (2022). A Review of Oxylipins in Alzheimer’s Disease and Related Dementias (ADRD): Potential Therapeutic Targets for the Modulation of Vascular Tone and Inflammation. Metabolites.

[47] Yamaguchi A., Botta E., Holinstat M. (2022). Eicosanoids in Inflammation in the Blood and the Vessel. Front. Pharmacol..

[48] Gullotta G.S., Costantino G., Sortino M.A., Spampinato S.F. (2023). Microglia and the Blood-Brain Barrier: An External Player in Acute and Chronic Neuroinflammatory Conditions. Int. J. Mol. Sci..

[49] Serhan C.N., Levy B.D. (2018). Resolvins in Inflammation: Emergence of the pro-Resolving Superfamily of Mediators. J. Clin. Investig..

[50] Dyall S.C., Balas L., Bazan N.G., Brenna J.T., Chiang N., da Costa Souza F., Dalli J., Durand T., Galano J.-M., Lein P.J. (2022). Polyunsaturated Fatty Acids and Fatty Acid-Derived Lipid Mediators: Recent Advances in the Understanding of Their Biosynthesis, Structures, and Functions. Prog. Lipid Res..

[51] Chistyakov D.V., Gavrish G.E., Goriainov S.V., Chistyakov V.V., Astakhova A.A., Azbukina N.V., Sergeeva M.G. (2020). Oxylipin Profiles as Functional Characteristics of Acute Inflammatory Responses in Astrocytes Pre-Treated with IL-4, IL-10, or LPS. Int. J. Mol. Sci..

[52] Lee J.Y., Han S.H., Park M.H., Song I.-S., Choi M.-K., Yu E., Park C.-M., Kim H.-J., Kim S.H., Schuchman E.H. (2020). N-AS-Triggered SPMs Are Direct Regulators of Microglia in a Model of Alzheimer’s Disease. Nat. Commun..

[53] Rowlinson S.W., Crews B.C., Goodwin D.C., Schneider C., Gierse J.K., Marnett L.J. (2000). Spatial Requirements for 15-(R)-Hydroxy-5Z,8Z,11Z,13E-Eicosatetraenoic Acid Synthesis within the Cyclooxygenase Active Site of Murine COX-2—Why Acetylated COX-1 Does Not Synthesize 15-(R)-Hete. J. Biol. Chem..

[54] Siskind L.J., Colombini M. (2000). The Lipids C2- and C16-Ceramide Form Large Stable Channels—Implications for Apoptosis. J. Biol. Chem..

[55] Liddelow S.A., Barres B.A. (2017). Reactive Astrocytes: Production, Function, and Therapeutic Potential. Immunity.

[56] Singh D. (2022). Astrocytic and Microglial Cells as the Modulators of Neuroinflammation in Alzheimer’s Disease. J. Neuroinflamm..

