# Supplementary material for: A Nominally Safe Dose of Fumonisins Induces Mild Neuroinflammation in Chickens by Targeting Sphingolipids and Oxylipins but Not Cytokines
Source: Antioxidants (Basel). 2026 Apr 25;15(5):546. doi: 10.3390/antiox15050546 (PMC13203082; doi:10.3390/antiox15050546)
Supplement: Supplementary file 1 [file antioxidants-15-00546-s001.zip › Sup_Figures.pdf]

Figure S1.

A

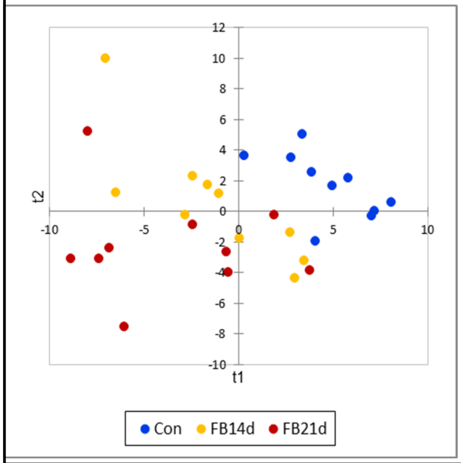

Model quality

Statistique	Comp1	Comp2	Comp3	Comp4
Q^2 cum	0.218	0.222	0.304	0.489
R^2Y cum	0.245	0.354	0.551	0.719
R^2X cum	0.427	0.639	0.735	0.795

Confiusion matrix

From \ To	Con	FB14d	FB21d	Total	% correct
Con	10	0	0	10	100%
FB14d	0	10	0	10	100%
FB21d	1	1	8	10	80%
Total	11	11	8	30	93%

B

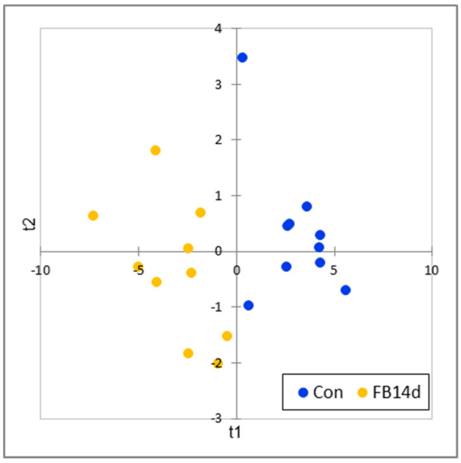

Model quality

Statistique	Comp1	Comp2	Comp3	Comp4
Q^2 cum	0.715	0.709	0.742	0.595
R^2Y cum	0.713	0.788	0.847	0.905
R^2X cum	0.583	0.706	0.789	0.801

Confusion matrix

From \ To	Con	FB14d	Total	% correct
Con	10	0	10	100%
FB14d	0	10	10	100%
Total	10	10	20	100%

C

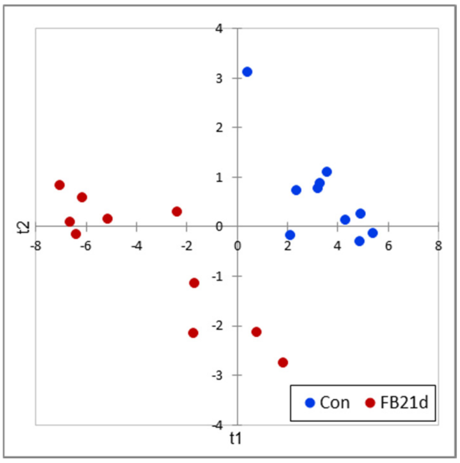

Model quality

Statistique	Comp1	Comp2	Comp3	Comp4
Q^2 cum	0.663	0.843	0.867	0.883
R^2Y cum	0.639	0.878	0.901	0.920
R^2X cum	0.653	0.718	0.824	0.868

Confusion matrix

From \ To	Con	FB21d	Total	% correct
Con	10	0	10	100%
FB21d	0	10	10	100%
Total	10	10	20	100%

Figure S1. PLS-DA analysis of the brain sphingolipid profile in chickens. The results were obtained by comparing the control group (Con) with chickens that were fed 14.6 mg FB1+FB2/kg for 14 days (FB14d) or 21 days (FB21d). The PLS-DA analysis was performed (B) between the control group and the FB14d group, and (C) between the control group and the FB21d group.

Figure S2.

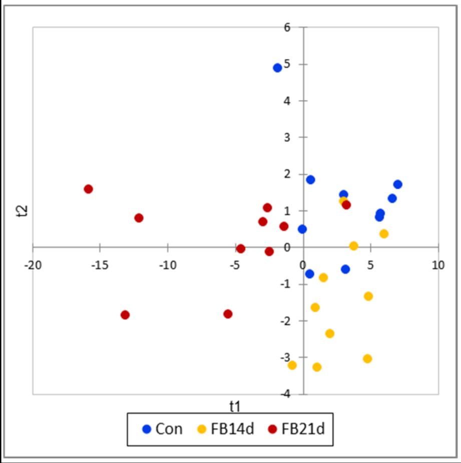

Model quality:

Statistique	Comp1	Comp2	Comp3	Comp4
Q² cum	0.253	0.369	0.344	0.373
R²Y cum	0.252	0.433	0.513	0.594
R²X cum	0.720	0.800	0.853	0.886

Confusion matrix

From \ To	Con	FB14d	FB21d	Total	% correct
Con	10	0	0	10	100%
FB14d	2	8	0	10	80%
FB21d	0	0	10	10	100%
Total	12	8	10	30	93%

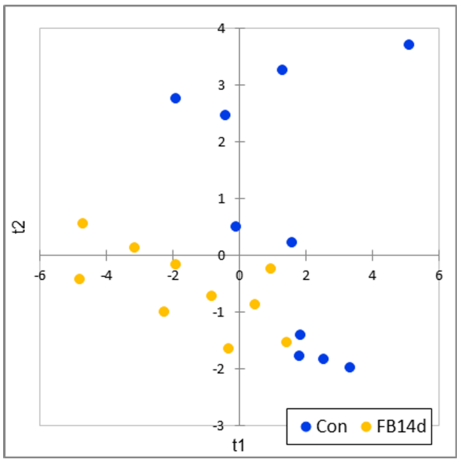

Model quality

Statistique	Comp1	Comp2	Comp3	Comp4
Q² cum	0.278	0.258	0.387	0.206
R²Y cum	0.343	0.457	0.626	0.683
R²X cum	0.364	0.646	0.744	0.802

Confusion matrix

From \ To	Con	FB14	Total	% correct
Con	10	0	10	100%
FB14	0	10	10	100%
Total	10	10	20	100%

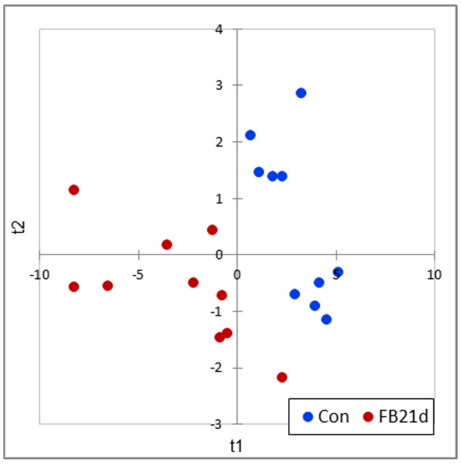

Model quality

Statistique	Comp1	Comp2	Comp3	Comp4
Q² cum	0.547	0.695	0.759	0.776
R²Y cum	0.539	0.721	0.804	0.871
R²X cum	0.739	0.830	0.870	0.882

Confusion matrix

From \ To	Con	FB21d	Total	% correct
Con	10	0	10	100%
FB21d	0	10	10	100%
Total	10	10	20	100%

Figure S2. PLS-DA analysis of the brain oxylipin profile in chickens. The results were obtained by comparing the control group (Con) with chickens that were fed 14.6 mg FB1+FB2/kg for 14 days (FB14d) or 21 days (FB21d). The PLS-DA analysis was performed (B) between the control group and the FB14d group, and (C) between the control group and the FB21d group.

Figure S3.

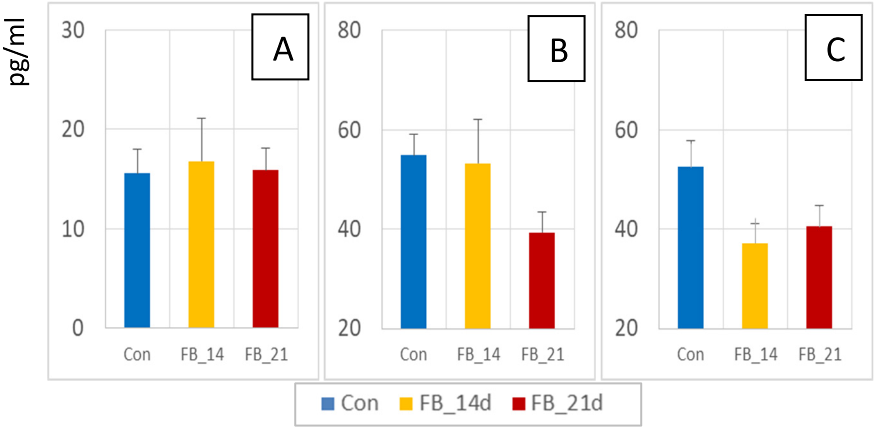

Figure S3. Concentrations of cytokines in the brains of chickens fed a control, mycotoxin-free diet (Con) or a diet containing 14.6 mg FB1+FB2/kg for 14 days (FB14) or 21 days (FB21): (A) TNFα, (B) IL-1β, and (C) IL-10. Values are expressed as mean ± SE (n = 10 per group). Differences between groups were assessed by ANOVA, with statistically significant differences (Tukey's test, $p < 0.05$) indicated by different letters.

Figure S4.

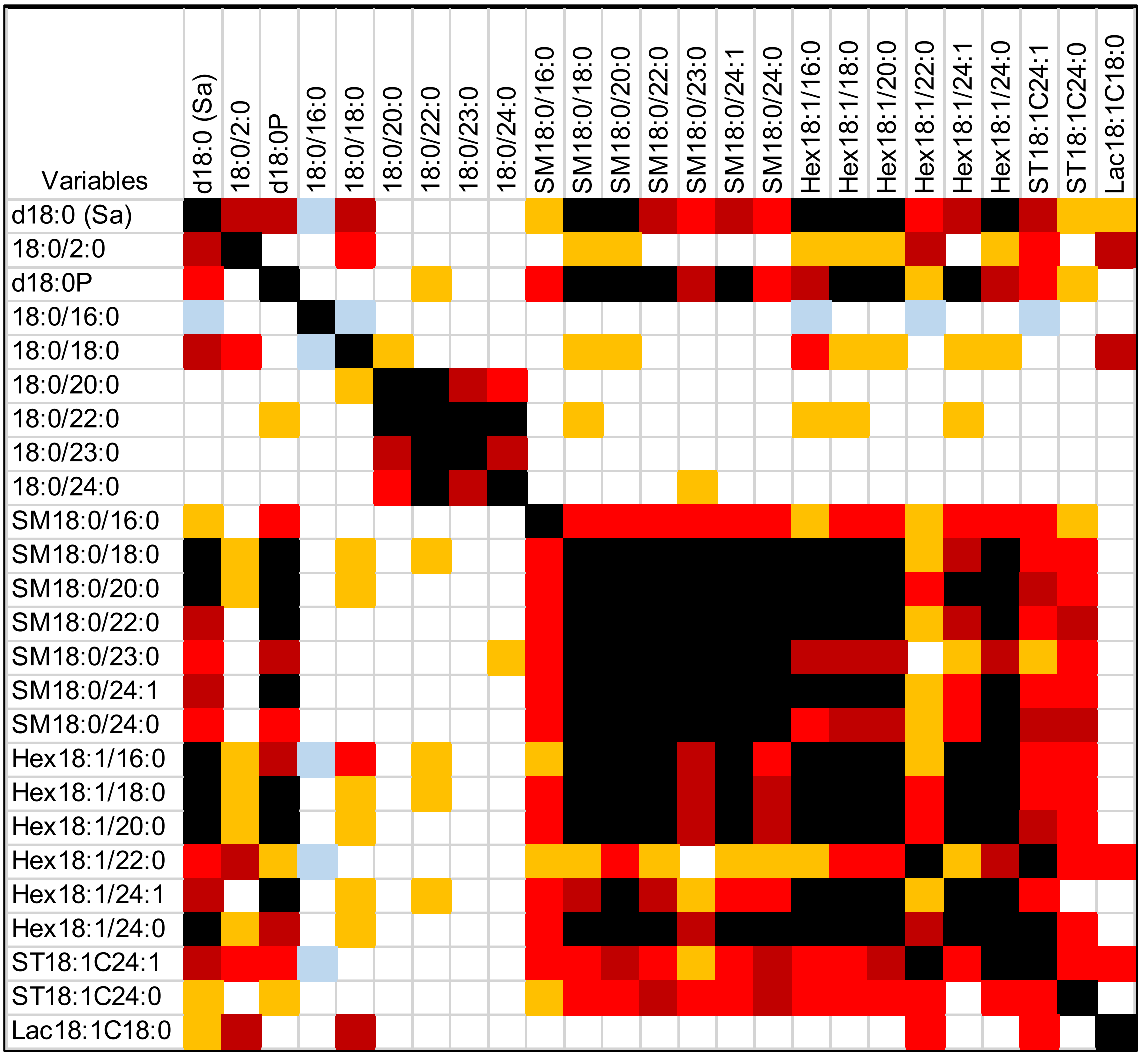

Figure S4. Correlation analyses between dihydrosphingolipids and glycosylceramides measured in the brains of chickens fed a control, mycotoxin-free diet or a diet containing 14.6 mg FB1+FB2/kg for 14 days or 21 days.
